# Mg doped SnO_2_ electron transport layer enhances planar all inorganic perovskite solar cells efficiency

**DOI:** 10.1016/j.isci.2025.113239

**Published:** 2025-07-29

**Authors:** Qixin Chen, Zhongchen Bai, Xu Wang, Xishun Peng, Xinghua Li, Cheng Zuo, Zhengping Zhang

**Affiliations:** 1 College of Big Data and Information Engineering, Guizhou University, Guiyang City 550025, China; 2 Guizhou Province Key Lab. for Photoelectric Technology and Application, Guizhou University, Guiyang City 550025, China; 3 Key Laboratory of Advanced Manufacturing Technology, Ministry of Education, Guiyang 550025, Guizhou, China; 4 Guizhou Polytechnic of Construction, Guiyang City 551499, China

**Keywords:** chemistry, physics, energy engineering, materials science

## Abstract

The electron transport layer (ETL) plays a crucial role in fabricating efficient and stable planar perovskite solar cells (PSCs). Among the various alternatives to TiO_2_ for electron transport layers (ETLs), tin oxide (SnO_2_) has emerged as a highly promising candidate due to its outstanding potential. However, improvements in SnO_2_ materials remain necessary due to inherent limitations, such as low conductivity, high energy barrier, and interfacial defects. In this study, Mg^2+^ ions were introduced into SnO_2_ to substitute Sn sites, leading to an increase in the open-circuit voltage (V_oc_) of PSCs, and consequently enhancing overall device efficiency. With a Mg doping concentration of 3.0%, the V_oc_ and power conversion efficiency (PCE) of the solar cell reached 1.540 V and 7.17%, respectively. The incorporation of Mg^2+^ ions into SnO_2_ presents an effective method to improve the performance of SnO_2_ ETL in PSCs while utilizing more environmentally friendly solvents.

## Highlights

• Mg doping (3.0%) in SnO_2_ ETL improves PSC PCE to 7.17% without HTL
• Enhanced charge transfer and reduced recombination at the ETL/perovskite interface
• Devices fabricated with green solvents show improved reproducibility and stability
• Achieved 32.6% efficiency gain and good ambient stability without encapsulation

## Graphical abstract

**Figure:**
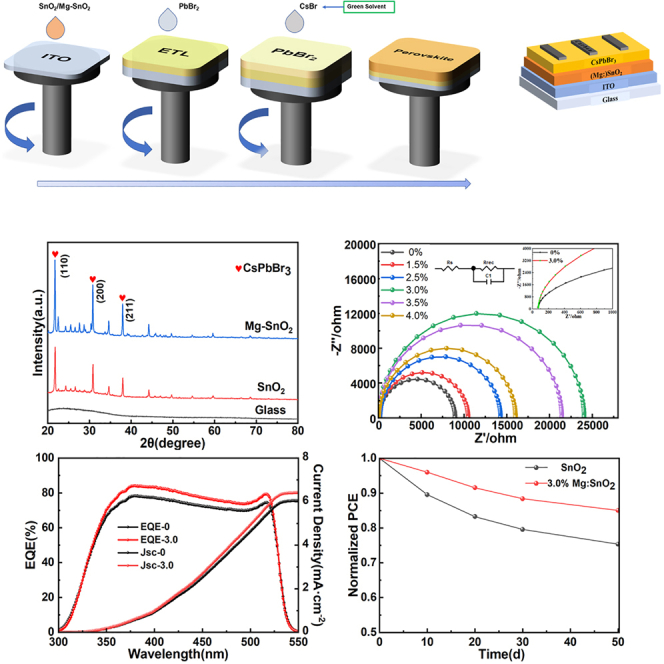

## Introduction

With the rising energy consumption and increasing global demand, pressure on energy resources has significantly intensified. Among renewable energy sources, solar energy is one of the most abundant, environmentally sustainable, and cost-effective options, making it a promising alternative. As a result, solar cells have gained recognition as a viable technology to mitigate the ongoing energy crisis. Advances in research and technological breakthroughs have led to a rapid enhancement in the power conversion efficiency (PCE) of perovskite solar cells (PSCs) increasing from 3.8%^1^ to 26.70%.^2^ Additionally, the PCE of tandem solar cells has surpassed 29.8%.^3^ Notably, organic-inorganic solar cells demonstrate outstanding photoelectric properties,^4^ including a tunable direct bandgap,^5^ extended carrier diffusion length,^6^ high absorption coefficient,^7^ and efficient carrier mobility.^8^ These attributes make them a key focus in the development of PSC technology.

The ETL serves as a critical component in planar PSCs,^9^ by enhancing carrier extraction speed and electron transport efficiency. Moreover, it plays a significant role in determining the nucleation quality of the perovskite layer during the growth process,^10^ directly influencing the overall performance of PSC devices. Commonly utilized ETL materials include TiO_2_,^11^ ZnO,^12^ and SnO_2_,^13^ which have attracted considerable research interest due to their low manufacturing costs and exceptional thermal stability.^4^ Among these materials, SnO_2_ is considered a promising candidate for the ETL in PSCs because of its favorable band alignment,^14^ wide bandgap, and high bulk carrier mobility.^9^^,^^15^^,^^16^ However, intrinsic limitations, such as severe charge recombination at the interfaces between contact layers,^17^ hinder its commercial viability and large-scale application. To mitigate these drawbacks, extensive research has been conducted to enhance its performance.

In 2013, Brion’s team utilized a solvothermal method to stabilize the gel dispersion of SnO_2_ nanoparticles within a size range of 2–4 nm at a reaction temperature of 150°C, achieving a PCE of 5.24%.^18^ In 2015, the Fang group employed a low-temperature solution treatment method, to introduce high-performance SnO_2_ nanocrystals into the ETL, ultimately improving the PCE to 17.21%.^4^^,^^19^ Likewise, Zhang and colleagues applied a plasma-enhanced atomic layer deposition technique to lower the SnO_2_ deposition temperature to 100°C, enabling the development of flexible devices with a maximum PCE of 16.08%.^20^

Despite these advancements, the utilization of SnO_2_ as an ETL continues to present challenges, including severe hysteresis, charge accumulation, and charge recombination at the interface with the perovskite layer.^21^ To address these issues, several strategies have been investigated, such as element doping,^22^ interface modification,^23^ and the incorporation of multi-layer ETL structures.^24^ These approaches have demonstrated significant potential in enhancing the performance of SnO_2_-based ETLs.

The Fang group developed gradient nano-SnO_2_ as the ETL using a low-temperature hydrothermal growth method (below 100°C). However, the resulting device exhibited severe hysteresis due to improper energy level alignment in this approach.^4^^,^^25^ To address this issue, Y was doped into the SnO_2_ ETL during the same growth process, leading to the formation of a well-ordered array of SnO_2_ nanosheets, which significantly reduced hysteresis. The device fabricated using this technique achieved a champion efficiency of 17.29%. This method enhanced the interfacial contact by expanding the contact area between the SnO_2_ ETL and the perovskite layer, thereby improving overall device performance.^4^^,^^26^

Research has also focused on the application of green solvents in PSCs, demonstrating promising results. For instance, Wang Shi-bo’s group achieved a PCE of 9.55% by substituting the toxic solvent methanol with green solvents in the perovskite layer.^27^ Furthermore, strategies involving two-layer ETL models, such as SnO_2_/TiO_2_,^28^ SnO_2_/In_2_O_3_,^29^ and SnO_2_/ZnO,^30^ have been investigated to enhance device efficiency. Nevertheless, element doping remains one of the most effective and cost-efficient approaches for optimizing energy level alignment and regulating carrier concentration.^31^ Commonly utilized doping elements include Li, Zn, Y, Nb, Al, and Mg.^4^

Doping Mg into SnO_2_ facilitates the replacement of Sn^2+^ ions,^32^ leading to an increase in hole concentration with higher Mg concentrations. He’s group investigated this newly formed P-type semiconductor through Mg doping, with experimental results confirming that the sample exhibited a high hole concentration.^32^ Additionally, Fang’s group introduced a novel approach by utilizing Mg-doped, high-temperature-treated quantum dot SnO_2_ as a barrier layer, along with a relatively thin SnO_2_ mesoporous layer, to enhance the PCE and overall performance of PSCs.^33^ However, these methods present certain limitations, including high costs and the requirement for advanced equipment, such as electron beam systems for evaporation and deposition. Moreover, in earlier studies, methanol, a toxic and hazardous solvent, was predominantly used for the inorganic perovskite layer.

Limited research has focused on improving SnO_2_ ETL through Mg doping while incorporating green solvents. Mg^2+^ was chosen as the doping ion because of its excellent lattice compatibility, effective regulation of electronic structure, significant interfacial optimization effects, and its comprehensive potential to enhance carrier transport properties and device stability. For green solvents, water exhibits strong solubility for CsBr,^34^ while ethylene glycol monomethyl ether (EGME) offers relatively high local viscosity and good solvation ability.^35^ The mixed solvent formed by combining the two helps to produce a homogeneous solution and evaporates more slowly than methanol, thereby promoting a more complete reaction between CsBr and PbBr_2_. Both water and EGME are low-toxicity or non-toxic solvents, and the spin-coating process requires fewer applications compared to CsBr solutions prepared with toxic methanol.^27^ This significantly reduces potential hazards to both the environment and laboratory personnel, offering improved environmental friendliness and operational safety. In terms of economic cost, both the material costs and usage-related expenses associated with water and EGME are lower than those of CsBr solutions formulated with conventional methanol.

**Figure 1:**
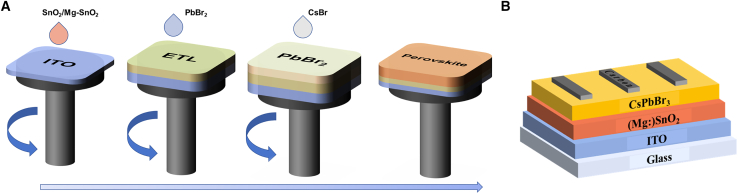
Schematic diagram illustrating the fabrication and structure of an all-inorganic perovskite solar cell (A) Schematic illustration of the spin-coating process for the fabrication of ETLs and CsPbBr_3_ films. (B) The device configuration of ITO/ETL/CsPbBr_3_/carbon.

In the present study, a high-performance ETL was developed for flat-plate all-inorganic PSCs by doping Mg into SnO_2_ solution, combined with the use of green solvents for treating the perovskite layer solution. This approach significantly enhanced the overall efficiency of inorganic PSCs with the structure of ITO/SnO_2_/CsPbBr_3_/carbon. As a result, the comprehensive performance of PSCs was notably improved. The spin-coating process and device structure are depicted in Figures 1A and 1B.

## Results and discussion

### Improved structural and electrical properties of SnO_2_ ETL through Mg doping

**Figure 2:**
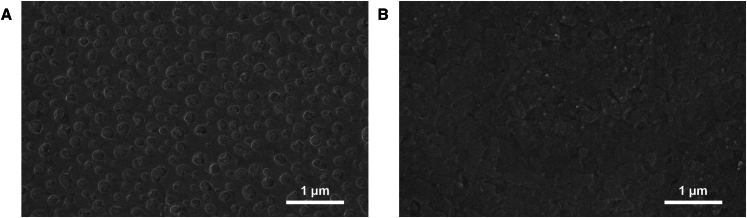
Top-view SEM images of ETLs (A and B) Top-view SEM images of SnO_2_ (A) and 3.0% Mg-doped SnO_2_ (B) films. Scale bar: 1 μm.

**Figure 3:**
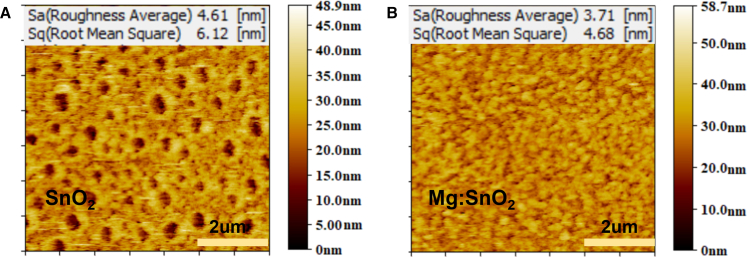
Effect of Mg doping on the surface morphology and roughness of SnO_2_ films (A) AFM topographic image of SnO_2_ film with a surface roughness average (Sa) of 4.61 nm. (B) AFM image of 3.0% Mg-doped SnO_2_ film showing a smoother surface with Sa of 3.71 nm. Scale bar: 2 μm.

**Figure 4:**
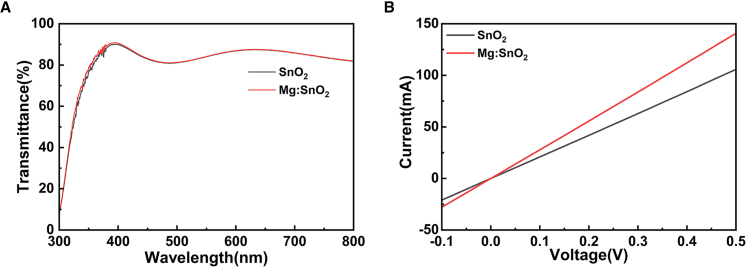
Mg doping significantly enhances the conductivity of SnO_2_ films while maintaining high transparency (A) Transmittance spectra of SnO_2_ and 3.0% Mg: SnO_2_ ETLs. (B) I-V curve of SnO_2_ and 3.0% Mg-doped SnO_2_ films.

The surface morphologies of SnO_2_ films deposited from SnO_2_ solution and 3.0% Mg-doped SnO_2_ solution on ITO substrates were analyzed using top-view SEM and atomic force microscopy (AFM) imaging, as presented in Figures 2 and 3, respectively. Figures 2A and 2B indicate that the SnO_2_ film exhibits uniformly distributed particles with similar particle sizes, whereas the Mg-doped SnO_2_ film presents a smoother surface (Figure S1). Furthermore, AFM characterization confirms that particle distribution in the 3.0% Mg-doped SnO_2_ film is more uniform, as shown in Figure 3. The surface roughness decreases from 4.61 nm in the SnO_2_ film to 3.71 nm in the 3.0% Mg-doped SnO_2_ film, with the root-mean-square (RMS) roughness reducing from 6.12 nm to 4.68 nm. These results suggest that Mg incorporation into SnO_2_ leads to a smoother surface morphology, which benefits the deposition of the perovskite film and promotes grain growth in the perovskite absorber layer. The characterization results further demonstrate that Mg doping facilitates the formation of a more uniform, dense, and smooth SnO_2_ film, thereby improving its quality on the ITO surface. This enhancement significantly increases the uniformity and surface coverage of the perovskite film, contributing to the deposition of a higher-quality perovskite layer on the SnO_2_ ETL.^31^ To examine changes in light transmission due to doping, transmittance measurements were conducted for SnO_2_ and 3.0% Mg-doped SnO_2_ films, as shown in Figure 4A. The results indicate that the 3.0% Mg-doped SnO_2_ films exhibit slightly enhanced transmittance compared to undoped SnO_2_ films within the 300–400 nm spectral range, improving optical properties and promoting greater light absorption in the perovskite layer. Additionally, Figure 4B demonstrates that the 3.0% Mg-doped SnO_2_ ETL possesses significantly higher conductivity, which directly contributes to enhanced current density performance in the devices.

### Elemental distribution in the ETL and optimization of CsPbBr_3_ composition

**Figure 5:**
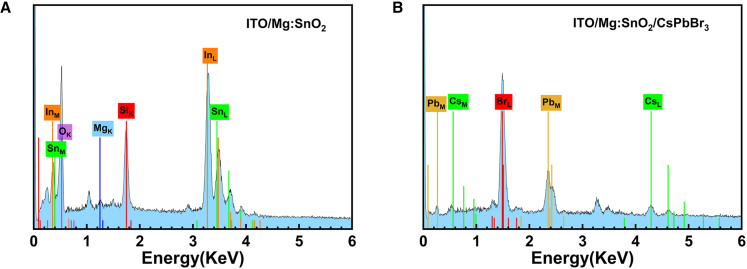
EDS spectra of ETLs (A and B) EDS spectra of Mg: SnO_2_ ETL (A) and ITO/Mg-doped SnO_2_/CsPbBr_3_ (B). The subscripts K, L, and M represent the characteristic X-ray emission series corresponding to each element.

EDS spectra and EDS mappings were utilized to assess elemental composition and spatial distribution in both Mg-doped and undoped SnO_2_ films, as illustrated in Figure 5 (Figures S2 and S3). The analysis confirms the presence and distribution of all constituent elements in the 3.0% Mg-doped SnO_2_ film, as shown in Figure 5A. Moreover, the findings reveal a uniform Mg distribution within the 3.0% Mg-doped SnO_2_ film. As shown in Figure 5B, the EDS spectrum of the CsPbBr_3_ film with 3.0% Mg doping displays the distribution of Cs, Pb, and Br. According to Figure S4 and Table S1, the atomic ratio of Cs, Pb, and Br in the absorption layer film at this doping level is close to 1:1:3, indicating an improved elemental distribution compared to the undoped condition. This result indicates that the primary phase of the CsPbBr_3_ film is the CsPbBr_3_ phase, with only minor amounts of impurity phases, such as CsPb_2_Br_5_ and Cs_4_PbBr_6_.^27^

### Enhancement of CsPbBr_3_ absorber layer quality

**Figure 6:**
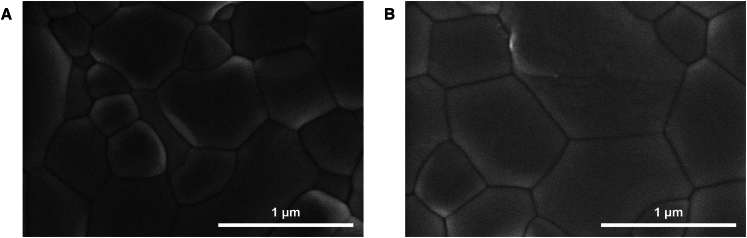
Top-view SEM images of CsPbBr_3_ perovskite films deposited on different ETLs (A and B) Top-view SEM images of CsPbBr_3_ perovskite films deposited on different ETLs: SnO_2_ (A) and 3.0% Mg-doped SnO_2_ ETLs (B). Scale bar: 1 μm.

**Figure 7:**
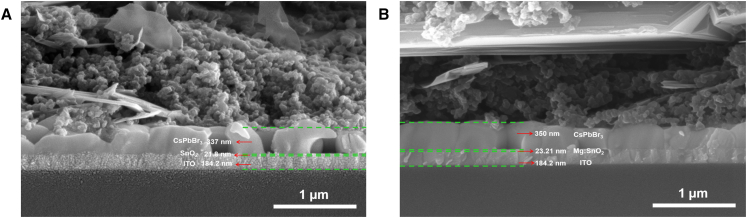
Cross-sectional SEM image of PSCs devices based on different ETLs (A and B) Cross-sectional SEM image of PSCs devices based on SnO_2_ (A) and 3.0% Mg-doped SnO_2_ ETLs (B). Scale bar: 1 μm.

The top-view SEM images of CsPbBr_3_ perovskite films and cross-sectional SEM images of PSC devices are presented in Figures 6 and 7 (Figure S5), respectively. Figures 6A and 6B illustrate the surface morphologies of perovskite films deposited on SnO_2_ ETL and 3.0% Mg-doped SnO_2_ ETL at identical magnification. The results indicate that the perovskite film deposited on the 3.0% Mg-doped SnO_2_ ETL exhibits notable improvements in morphological characteristics, including larger grain size, increased film density, and enhanced uniformity. These structural enhancements contribute to the reduction of carrier recombination at grain boundaries, suggesting that Mg doping facilitates the formation of a more uniform and compact SnO_2_ ETL on the ITO substrate during fabrication. The observed enhancement in device performance can be primarily attributed to the improved surface coverage characteristics, which align with previously reported findings in the literature.^36^ Experimental results confirm that the 3.0% Mg-doped SnO_2_ ETL provides a more favorable substrate for perovskite crystal growth. Cross-sectional analysis in Figure 7A reveals that the perovskite film deposited on undoped SnO_2_ ETL exhibits smaller grain dimensions in the vertical direction and larger inter-grain gaps, which impede the vertical transport of charge carriers. In contrast, as shown in Figure 7B, the perovskite film grown on the 3.0% Mg-doped SnO_2_ ETL demonstrates larger grain dimensions in the vertical direction, more compact inter-grain contact, and reduced voids. These characteristics enhance the vertical transport of charge carriers while mitigating non-radiative electron recombination, leading to improved electron transport efficiency.^4^ Although Mg doping in SnO_2_ can improve the surface quality of the ETL and facilitate the deposition and growth of the absorber layer, it may also affect the grain growth rate in certain local regions of the absorber. Additionally, despite the improved ETL quality due to Mg doping, the roughness and uneven morphology of the ITO substrate may hinder uniform film spreading of the ETL, resulting in non-uniform coverage in some areas. These factors can lead to the formation of voids during the deposition and growth of the absorber layer, which may adversely impact device performance.

### Crystallinity improvement in ETL and CsPbBr_3_ absorber layers

**Figure 8:**
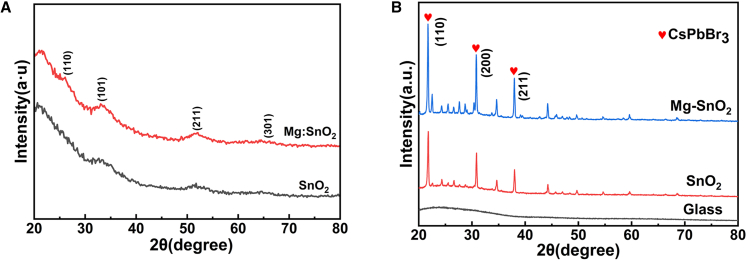
Mg doping improves the crystallinity of both SnO_2_ and CsPbBr_3_ layers (A) Grazing incidence XRD patterns of SnO_2_ and 3.0% Mg-doped SnO_2_ films spin-coated on quartz substrates. (B) XRD patterns of CsPbBr_3_ perovskite films deposited on SnO_2_ and 3.0% Mg-doped SnO_2_ ETLs coated on glass substrates.

In this study, considering that the SnO_2_ and Mg: SnO_2_ films were relatively thin and susceptible to interference from the ITO substrate,^4^^,^^37^ conventional XRD measurements were insufficient to effectively resolve their characteristic peaks. Therefore, grazing incidence X-ray diffraction (GIXRD) was employed to analyze the SnO_2_ and Mg: SnO_2_ films spin-coated on quartz substrates, as shown in Figure 8A. The diffraction peaks observed in the GIXRD patterns are in good agreement with the standard JCPDS card for SnO_2_ (No. 46–1088). Moreover, the Mg: SnO_2_ film exhibits slightly stronger diffraction peaks compared to the undoped SnO_2_ film. To avoid the interference of diffraction peak overlap caused by the ITO substrate, which may affect the accurate assessment of the crystallization behavior of the CsPbBr_3_ film, we performed measurements on a glass substrate. Specifically, two structures—glass/SnO_2_/CsPbBr_3_ and glass/3.0% Mg:SnO_2_/CsPbBr_3_—were fabricated. The crystalline properties of CsPbBr_3_ perovskite films deposited on SnO_2_ and 3.0% Mg-doped SnO_2_ films were further examined using XRD analysis, as depicted in Figure 8B. The comparative analysis reveals that the CsPbBr_3_ perovskite film deposited on the 3.0% Mg-doped SnO_2_ film exhibited a more intense diffraction peak than that observed for the film deposited on SnO_2_. This indicates an enhancement in the crystallinity of the CsPbBr_3_ perovskite.^4^

**Figure 9:**
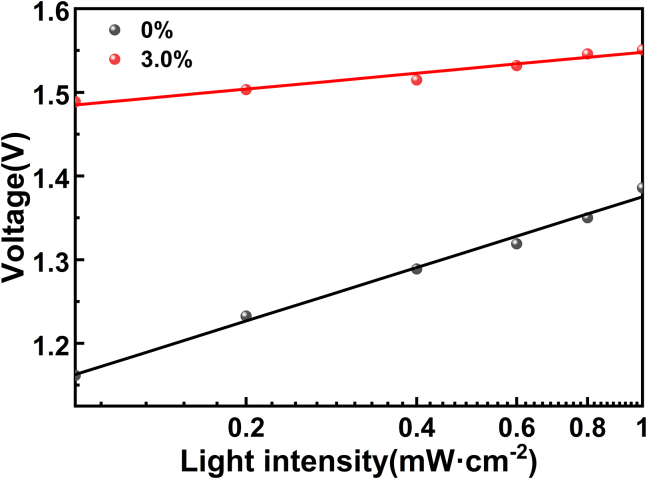
The dependence of Voc on light intensity for PSCs based on SnO_2_ and 3.0% Mg-doped SnO_2_ ETLs

According to the equation Voc = nkTln (I)/q + constant, the ideality factor (n) can be determined from the fitted curves of Voc obtained at varying simulated sunlight intensities (I), providing insight into trap-assisted Shockley-Read-Hall (SRH) recombination within the devices. Here, T represents absolute temperature, k is the Boltzmann constant, and q is the basic charge.^4^ As shown in Figure 9, the PSCs device with 3.0% Mg-doped SnO_2_ ETL had a lower ideality factor (n) (1.38) than the SnO_2_-based device (1.44), implying that SRH recombination and trap state were effectively suppressed in the PSCs device based on 3.0% Mg-doped SnO_2_ ETL.

### Enhanced carrier transport and suppression of non-radiative recombination

**Figure 10:**
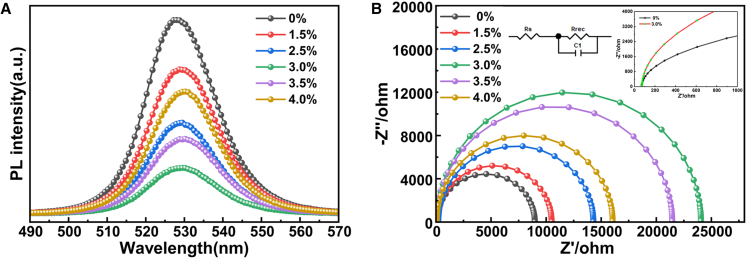
Optical and electrical characteristics of CsPbBr_3_ films and PSCs based on ETLs with different Mg doping concentrations (A) PL spectrum of perovskites deposited on SnO_2_ and Mg-doped SnO_2_ ETLs. (B) Nyquist plots of PSCs based on CsPbBr_3_ films deposited on SnO_2_ and Mg-doped SnO_2_ ETLs measured under dark conditions.

At the same time, steady-state photoluminescence (PL) and electrochemical impedance spectroscopy (EIS) measurements were performed, as shown in Figures 10A and 10B. Figure 10A illustrates that fluorescence emission of the perovskite film occurs at 528 nm, with the most pronounced PL quenching observed in the ITO/3.0% Mg-doped SnO_2_/perovskite configuration. This phenomenon is attributed to enhanced electron transfer at the SnO_2_/perovskite interface.^4^ Experimental data in Figure 10A indicate that a 3.0% Mg doping ratio effectively suppresses carrier recombination at the interface while maximizing the carrier extraction rate. As the Mg doping concentration increases, the PL quenching rate initially rises before slowing down, suggesting that complex impedance initially increases and subsequently decreases with a higher doping ratio. The changes in PL are mainly reflected in two aspects. On the one hand, Mg doping alters the quality and energy band structure of the ETL, thereby affecting the interfacial contact between the ETL and the absorber layer, which in turn influences the band alignment between them.^4^^,^^31^^,^^38^ Additionally, Mg-doped ETLs may impact crystal defects and carrier concentration, all of which together contribute to the observed shift in the PL peak position along the wavelength axis. On the other hand, Mg doping enhances the conductivity and electron mobility of the ETL and improves the interfacial contact between the ETL and the absorber layer. These improvements lead to more efficient carrier transport and reduced interfacial recombination, ultimately resulting in a decreased PL peak intensity.

Additionally, EIS measurements were conducted to analyze carrier migration and recombination behavior in PSCs. Figure 10B presents the Nyquist plot and the corresponding equivalent circuit diagram, obtained by fitting the data measured under dark conditions at a 1V bias using an electrochemical workstation. The plot shows a single semicircular feature, which is typically associated with the charge recombination process occurring in the low-frequency region. The semicircle radius represents charge recombination resistance (R_rec_), indicating the ease or difficulty of charge recombination.^27^ The series resistance (Rs) in the fitted circuit remained nearly constant across devices. Among all tested samples, the 3.0% Mg-doped device exhibited the largest semicircle radius, suggesting that this material has the best impedance characteristics and the charge recombination is most effectively suppressed. This result suggests the most effective suppression of charge recombination within the solar cell, minimizing non-radiative recombination at the ETL/perovskite interface and significantly enhancing electron extraction efficiency. This, in turn, contributed to the enhancement of the device’s Voc and Fill factor (FF). The result is consistent with the PL measurements obtained in the experiment.^4^

To further distinguish between enhanced electron transport and defect-induced non-radiative recombination mechanisms, we conducted time-resolved photoluminescence (TRPL) measurements and electrochemical trap-state density analyses, as shown in Figure S6 and Table S2. The analysis results indicate that the TRPL data (Figure S6A and Table S2) reveal a significantly increased carrier injection rate from the perovskite layer into the ETL, with interfacial non-radiative recombination channels effectively suppressed, thereby leading to an evident improvement in overall carrier separation efficiency. The electrochemical trap-state analysis (Figure S6B) demonstrates that the 3.0% Mg: SnO_2_ sample exhibits the lowest trap-state response across the entire frequency range, suggesting that Mg doping effectively reduces the interfacial defect density and consequently minimizes non-radiative recombination. Combined with previous characterizations including EIS, PL, and SEM, these results comprehensively verify the multiple advantages of the 3.0% Mg-doped SnO_2_ ETL in enhancing electron injection, suppressing non-radiative recombination, and improving film morphology, thereby further confirming its positive impact on the overall device performance.

### Improvement in photovoltaic performance of the device

**Figure 11:**
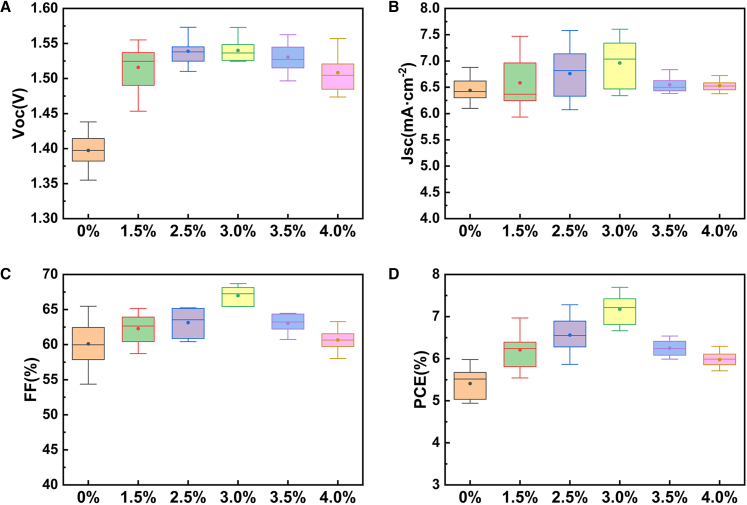
The photovoltaic parameters for PSC samples based on different ETLs Doping ratio-dependent statistical photovoltaic parameters of (A) Voc, (B) Jsc, (C) FF, and (D) PCE. Each boxplot represents the interquartile range (IQR), the center line indicates the median, and the whiskers denote the minimum and maximum values. A total of 10 independent devices (*n* = 10) were measured for each doping ratio.

The optimal doping ratio of 3.0% was determined through J-V characterization under AM 1.5G simulated sunlight illumination (100 mW/cm^2^) in reverse scan, as illustrated in Figure 11. Devices utilizing 3.0% Mg-doped SnO_2_ ETL demonstrated an improved average PCE of 7.17%, representing an approximate 32.63% enhancement compared to the reference device, which exhibited an average PCE of 5.41%, as shown in Figure 11 and Table S3. According to Table S3, the V_oc_ increased from 1.397 V to 1.540 V, the FF improved from 60.11% to 66.98%, and the Short-circuit current density (Jsc) increased from 6.440 mA cm^−2^ to 6.962 mA cm^−2^. The SEM and AFM characterizations of the ETL layer show that the SnO_2_ ETL formed with 3.0% Mg doping exhibits a denser, more uniform, and smoother film morphology. Meanwhile, results from EIS measurements and electrochemical defect density analysis indicate that the ETL with 3.0% Mg doping effectively reduces non-radiative recombination at the ETL/absorber interface. PL measurements further confirm that the electron transport efficiency is significantly enhanced in samples fabricated on the ETL with 3.0% Mg doping. In addition, J–V test results across different doping concentrations demonstrate that the 3.0% doping level yields the best performance in all key photovoltaic parameters, indicating that 3.0% is an optimal doping concentration.

We conducted repeatability experiments on devices fabricated with SnO_2_ ETLs doped with different concentrations of Mg, increasing the number of samples in each group from 10 to 20. The statistical results from the repeated experiments showed that the variations in key parameters, including PCE, V_oc_, FF, and Jsc, remained within a reasonable range, demonstrating good reproducibility and stability, as shown in Figure S7 and Table S4. However, a noticeable hysteresis effect remains in these devices, as shown in Figure S8.

**Figure 12:**
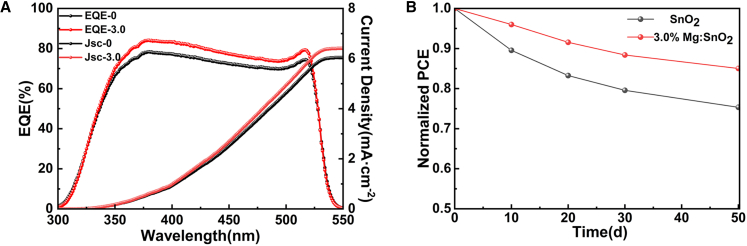
Mg doping improves the photoresponse and long-term stability of PSCs in ambient conditions (A) EQE spectra and integrated current of PSCs based on SnO_2_ and 3.0% Mg-doped SnO_2_ ETLs. (B) Stability measurement of PSCs devices fabricated with SnO_2_ and 3.0% Mg-doped SnO_2_ ETLs without encapsulation in air atmosphere at 20°C and 60% RH.

Based on previous studies, the primary causes of hysteresis in planar SnO_2_-based PSCs include: (1) energy level mismatch at the SnO_2_/perovskite interface^23^^,^^24^; (2) unbalanced carrier transport, where hole transport is significantly faster than electron transport due to hole extraction and injection.^4^ This study adopts an HTL-free device structure, using carbon electrodes as the hole-collecting contact. Since the carbon electrode physically contacts the perovskite and lacks selective hole extraction capability, the overall device may still exhibit hysteresis, even if Mg doping enhances electron extraction efficiency, unless the hole transport pathway is simultaneously optimized; and (3) intrinsic ion defects within perovskite films, which provide pathways for ion migration.^39^ Perovskite is a “soft ionic crystal” that contains a large number of mobile ions.^4^^,^^39^ Under an applied bias, these ions may migrate toward the interface, causing local potential fluctuations and instability in interfacial energy level alignment. This can lead to an asymmetric energy band structure, which ultimately manifests as a hysteresis effect. This ion migration mechanism is an intrinsic property of perovskite materials and cannot be fully eliminated by solely modifying the ETL. Furthermore, defect accumulation on the SnO_2_ surface contributes to electron trapping during the low-temperature process, hindering carrier transport and exacerbating hysteresis.^4^^,^^40^ Although Mg doping improves the crystallinity and surface smoothness of SnO_2_, the low-temperature solution-based preparation of SnO_2_ inevitably results in a high density of surface defect states.^4^^,^^23^^,^^40^^,^^41^ These defects make it difficult to fundamentally eliminate lattice mismatch and defect accumulation at the SnO_2_/perovskite interface. In this study, apart from environmental variations, the SnO_2_ solution preparation method used was consistent with the method reported by the Fang group. This preparation technique, due to its surface characteristics, exhibits considerable hysteresis, a phenomenon also observed in previous research.^4^^,^^25^^,^^31^ Several studies have reported that PSCs without hysteresis effects can be achieved using an interface modifier between the SnO_2_ ETL and the perovskite film.^25^^,^^42^ While metal doping in SnO_2_ enhances conductivity and energy level alignment, its impact on reducing hysteresis remains limited, as it fails to eliminate surface defect states in SnO_2_ and perovskite or resolve the lattice matching issue at the ETL/perovskite interface.^4^^,^^21^^,^^31^ Consequently, since no additional interface treatment was performed in this study, the J-V test results still exhibit evident hysteresis. However, further research is ongoing. EQE tests were conducted on PSC devices incorporating SnO_2_ and Mg-doped SnO_2_ ETLs, as shown in Figure 12A and Figure S9. The EQE spectra and the corresponding integrated Jsc values for devices based on SnO_2_ and 3.0% Mg-doped SnO_2_ ETLs are presented in Figure 12A. The results indicate a significant enhancement in the EQE spectra of samples based on SnO_2_ and 3.0% Mg-doped SnO_2_ ETLs, with the calculated integrated Jsc values recorded at 6.04 and 6.41 mA cm^−2^, respectively. The increase in EQE values suggests that the doping method effectively improved the absorption efficiency of the perovskite layer, thereby enhancing its energy output performance. Absorption spectral measurements for ITO/SnO_2_/CsPbBr_3_ and ITO/3.0% Mg-doped SnO_2_/CsPbBr_3_ architectures exhibited strong alignment with the corresponding EQE profiles, as depicted in Figure S10. The suppression of carrier recombination in the Mg-doped SnO_2_ ETL and the improved quality of the perovskite film deposited on the Mg-doped SnO_2_ ETL contributed to the enhancement of Jsc and FF values to varying extents.

Stability is crucial parameter for evaluating device performance and remains one of the most significant challenges affecting the long-term reliability of PSCs.^4^^,^^42^ Stability testing was conducted by placing unencapsulated devices in a storage box, with the results presented in Figure 12B. After about 50 days of storage, the device incorporating a 3.0% Mg-doped SnO_2_ ETL demonstrated superior resistance to degradation, retaining 85.04% of its initial efficiency, whereas the device utilizing a SnO_2_ ETL maintained only 75.34% of its initial efficiency. These findings suggest that the device based on the 3.0% Mg-doped SnO_2_ ETL exhibits enhanced stability. This improvement is likely attributed to the combined effect of the optimized ETL and the use of environmentally friendly solvents during device fabrication, which collectively contribute to the improved crystallization of the perovskite layer. Similar findings have been reported in previous studies.^4^

## Discussion

In summary, the feasibility of utilizing environmentally friendly solvents for perovskite layer fabrication was explored, leading to the successful development of a method to enhance device performance by doping Mg into SnO_2_ ETL at low temperatures. This approach was established through the “green solvent addition + Mg doping” research model. Characterization and analysis results indicate that this method effectively increased the grain size of ETL films, thereby improving their overall quality, enhancing electron extraction and charge transport, and significantly reducing carrier recombination at the ETL/perovskite interface. Additionally, the light absorption properties of perovskite films were enhanced. Findings based on this research model suggest that the increased use of green solvents not only minimizes environmental impact during production but also lowers device fabrication costs by employing low-temperature doping techniques. These insights provide valuable guidance for the future commercialization of planar all-inorganic PSCs.

### Limitations of the study

The comparative effect in this study is limited. It focuses solely on investigating the influence of Mg doping in the electron transport layer under the condition of using a green solvent as the CsBr solvent, without conducting a comparison under the condition of using conventional methanol as the solvent.

## Resource availability

### Lead contact

Further information and requests for resources and reagents should be directed to and will be fulfilled by the lead contact, Zhengping Zhang (zpzhang@gzu.edu.cn).

### Materials availability

This study did not generate new unique reagents.

### Data and code availability

• The J–V performance parameters of the triple-cation devices are presented in Table S3.
• This study did not involve the development of entirely new original code.
• Any additional information required to reanalyze the data reported in this paper is available from the lead contact upon request.

## Acknowledgments

These works were supported by the 10.13039/501100001809National Natural Science Foundation of China (NSFC) (62065002); Guizhou Province High level Talent Training Plan (100 Levels) (no. QKHPTRC-GCC[2023]089); 10.13039/501100005329Guizhou Province Natural Science Foundation (Qiankehe Fundamentals – ZK[2024]General-106); Graduate Research Foundation of Guiyang University 2023 (no. 2023-YJS04).

## Author contributions

Conceptualization, Q.C., Z.B., and Z.Z.; methodology, Q.C. and Z.B.; investigation, Q.C., Z.B., and X.L.; writing—original draft, Q.C.; writing—review & editing, Q.C., Z.B., and C.Z.; funding acquisition, Z.Z. and Z.B.; resources, Z.Z., Z.B., and X.W.; software guidance, X.P.

## Declaration of interests

The authors declare no competing interests.

## STAR★Methods

### Key resources table

**Table:** 

REAGENT or RESOURCE	SOURCE	IDENTIFIER
**Chemicals, peptides, and recombinant proteins**		

2-Methoxyethanol	Macklin	CAS:109-86-4
Tin(II) chloride (SnCl_2_)	Macklin	CAS:7772-99-8
Magnesium chloride (MgCl_2_)	Macklin	CAS:7786-30-3
Thiourea	Macklin	CAS:62-56-6
cesium bromide(CsBr)	Aladdin	CAS:7787-69-1
lead bromide(PbBr_2_)	Macklin	CAS:10031-22-8
N, N-dimethylformamide (DMF)	Aladdin	CAS:68-12-2
ITO	HNXCKJ	https://yjigewxf1024.taobao.com/

### Method details

#### Preparation of SnO_2_ solution

A total of 0.853 g of SnCl_2_ and 0.285 g of thiourea were combined in a 150 mL Erlenmeyer flask along with 30 mL of deionized water. MgCl_2_ was then introduced in varying molar concentrations (0%, 1.5%, 2.5%, 3.0%, 3.5%, and 4.0%). The mixture underwent continuous stirring for about 24 h to ensure complete dissolution and reaction completion. The resulting SnO_2_ solutions were labeled as 0% SnO_2_, 1.5% SnO_2_, 2.5% SnO_2_, 3.0% SnO_2_, 3.5% SnO_2_, and 4.0% SnO_2_. Upon completion of the stirring process, the solutions were filtered using a 45 μm filter. The obtained filtrates were then sealed and stored for subsequent experiments.

#### Preparation of PbBr_2_ solution

A total of 2.018 g of PbBr_2_ powder was dissolved in 5 mL of DMF solvent within a 20 mL solution bottle. The bottle was placed in a water bath maintained at 80°C and stirred until the PbBr_2_ powder was fully dissolved, forming a 1.1 M PbBr_2_ solution. The solution was subsequently filtered using a 22 μm filter before being placed on a 90°C annealing platform for heating and storage at a constant temperature.

#### Preparation of CsBr solution

A total of 2.012 g of cesium bromide powder was added to a 20 mL solution bottle containing 5 mL of 2-methoxyethanol and 5 mL of deionized water. The mixture was stirred at room temperature for 1–2 h until complete dissolution. The solution was then filtered using a 45 μm filter, sealed, and stored at room temperature for further experiments.

#### Device fabrication

In this study, the conventional two-step spin-coating method was utilized to fabricate the CsPbBr_3_ perovskite absorber layer. Initially, the ITO substrate was subjected to UV ozone treatment for 15 min to improve surface wettability. Following this, 200 μL of SnO_2_ solution with varying Mg doping concentrations was spin-coated onto the ITO substrate at 3000 rpm for 30 s. The coated substrate was then transferred to a heating platform and annealed at 200°C for 1 h. After annealing, the substrate underwent a second UV ozone treatment for 10 min to enhance the wettability of the SnO_2_ surface.

Subsequently, 100 μL of PbBr_2_ solution was spin-coated onto the substrate at 2000 rpm for 30 s at 90°C for 30 min. Then, 115 μL of CsBr solution was spin-coated onto the PbBr_2_ film at 3000 rpm for 30 s and the substrate was transferred to an annealing platform at 250°C for 5 min. After annealing, it was removed from the platform and left to cool to room temperature. The spin-coating process is depicted in Figure 1A. A carbon scraper mask was then used to deposit the carbon electrode onto the ITO/SnO_2_/CsPbBr_3_ structure. The substrate was subsequently placed on an annealing platform at 110°C for about 10–15 min to ensure complete drying of the carbon electrode. Upon completion, the substrate was removed and allowed to cool to room temperature, resulting in a fully fabricated device with an active area of 0.12 cm^2^.

All preparation steps were performed entirely in an open-air environment, without any environmental control measures.

#### Device characterization

The surface morphology of thin films was examined using atomic force microscopy (AFM, CSPM5500, Ben Yuan). The photoluminescence (PL) spectra of perovskite films were recorded with a fiber optic spectrometer (QE6500, Ocean Optics) using a 355 nm wavelength laser (0355–05–01–0020–700, Cobolt Zouk) as the excitation source. The absorption spectrum of the sample was obtained using an integrating sphere spectrophotometer (HITACHI U–4100). The transmittance spectra of the electron transport layers (ETLs) were measured using a HITACHI UV-Vis-NIR spectrophotometer (UH4150) to evaluate their optical properties over a wide wavelength range. The surface morphology of the perovskite film and the cross-sectional crystalline structure of the device were examined using scanning electron microscopy (SEM, HITACHI, SU8010). EDS images were captured using Thermo Fisher Scientific FIB-SEM GX4 electron microscope at 10 kV and 43 pA operating Currents. The current density versus voltage (J-V) characteristic curves of the solar cells were plotted by the simulated solar illumination (SS-F5-3A, Enlitech) with a power standard of AM1.5 (100 mW/cm^2^). External quantum efficiencies (EQE) were measured using a quantum efficiency system (Enlitech QE–R 3011) in the air. The electrochemical impedance spectroscopy (EIS) of the device was measured using an electrochemical workstation (Vantone, AUTOLAB PGSTAT302N) in the dark state, in the range of 1 Hz–0.1 MHz. An X-ray diffractometer (XRD, UIV model, Rigaku; Rigaku Smart-Lab) was employed to characterize the crystallinity of different electron transport layers (ETLs) and the CsPbBr_3_ perovskite films deposited on them, respectively. Time-correlated singlephoton counting (TCSPC) by using a nanosecond-pulsed LED source (376 nm, FWHM ca. 600 ps) with a 40 MHz repetition rate was employed to measure the fluorescence–time profiles. A photomultiplier tube and a counting board (PicoQuanta, PicoHarp 300, Germany) were used for signal detection.

### Quantification and statistical analysis

In this study, statistical methods were primarily used to analyze the J–V performance parameters and their reproducibility, with a particular focus on the average values of these parameters. In addition, a basic quantitative and statistical analysis was conducted to evaluate the atomic ratios of Cs, Pb, and Br in the CsPbBr_3_ absorption layer, and the grain size distribution in ETL was also assessed.
