# Supplementary material for: Mg doped SnO2 electron transport layer enhances planar all inorganic perovskite solar cells efficiency
Source: iScience. 2025 Jul 29;28(9):113239. doi: 10.1016/j.isci.2025.113239 (PMC12362023; doi:10.1016/j.isci.2025.113239)
Supplement: Document S1. Figures S1–S10 and Tables S1–S4 [file mmc1.pdf]

iScience, Volume *28*

Supplemental information

Mg doped SnO_2 electron transport layer enhances planar all inorganic perovskite solar cells efficiency

Qixin Chen, Zhongchen Bai, Xu Wang, Xishun Peng, Xinghua Li, Cheng Zuo, and Zhengping Zhang

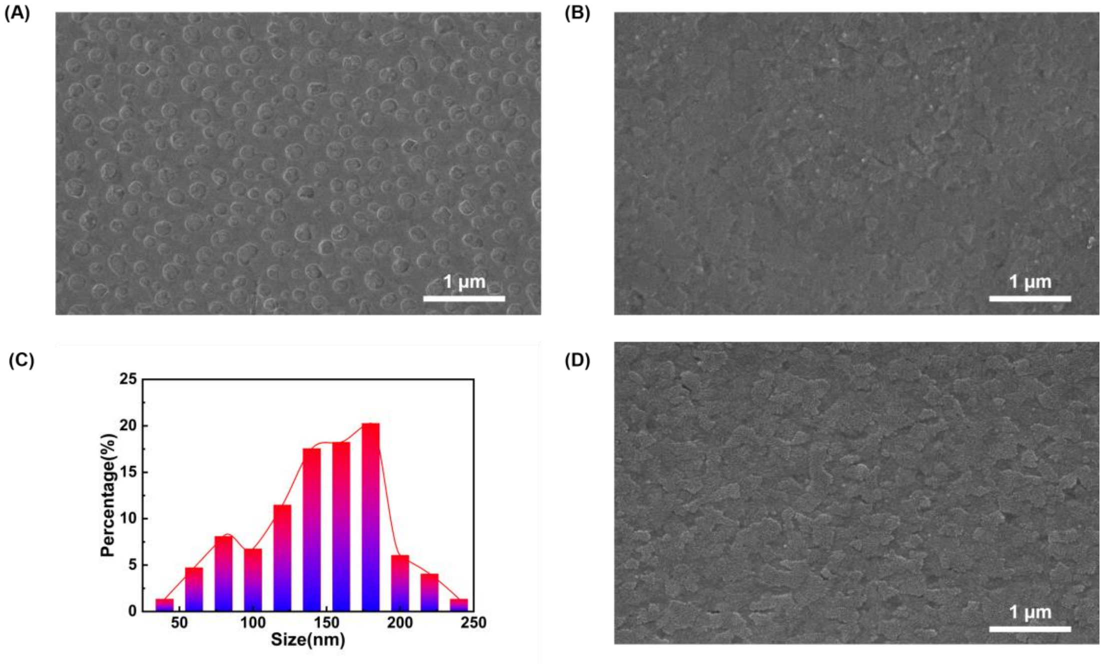

Figure S1 Top-view SEM images of (A) SnO_2 and (B) 3.0% Mg-doped SnO_2 films, (C)Histograms of grain sizes and (D)Top-view SEM image of bare ITO, related to Figure 2. Grain sizes were measured from SEM images, and a total of 202 grains were analyzed. Each bar represents the number of grains within the specified size range. The connecting line was added to visually indicate the trend across size intervals and does not represent an error measure. Scale bar: 1 μm.

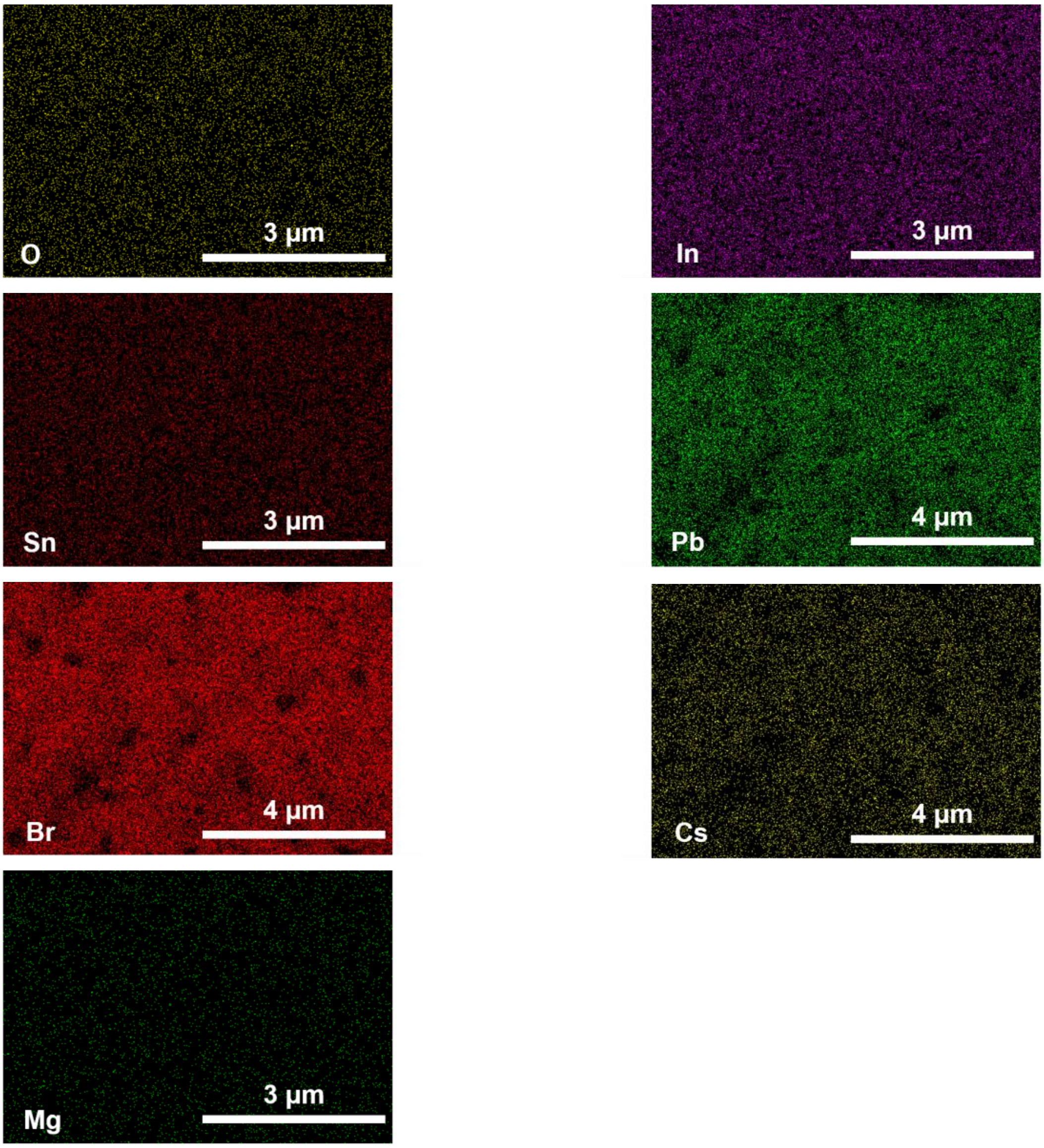

Figure S2 EDS mappings of Mg-doped SnO_2 ETL sample, related to Figure 5. Elemental mappings of O, Sn, Mg, and In were acquired with a scale bar of 3 μm. Elemental mappings of Br, Pb, and Cs were acquired with a scale bar of 4 μm.

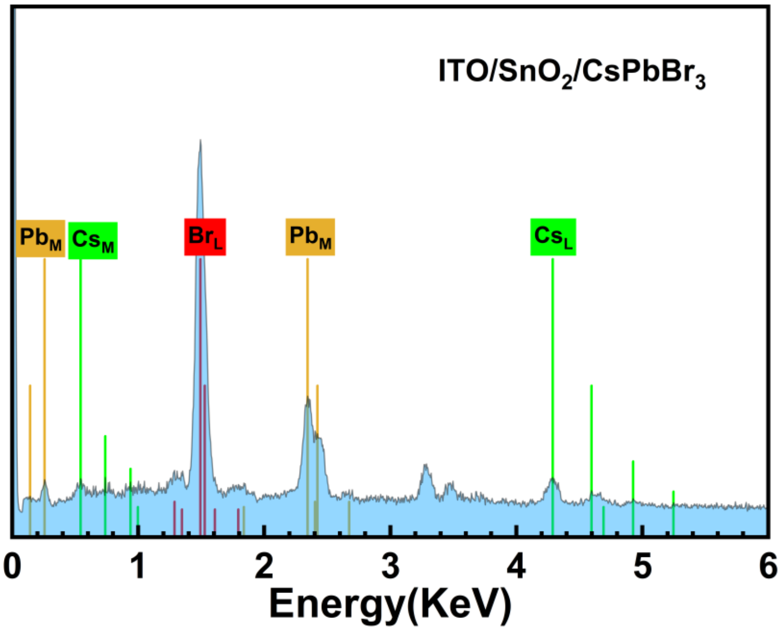

Figure S3 EDS spectrum of the ITO/SnO_2/$CsPbBr_3$ film, showing the characteristic peaks corresponding to Cs, Pb, and Br elements, related to Figure 5.

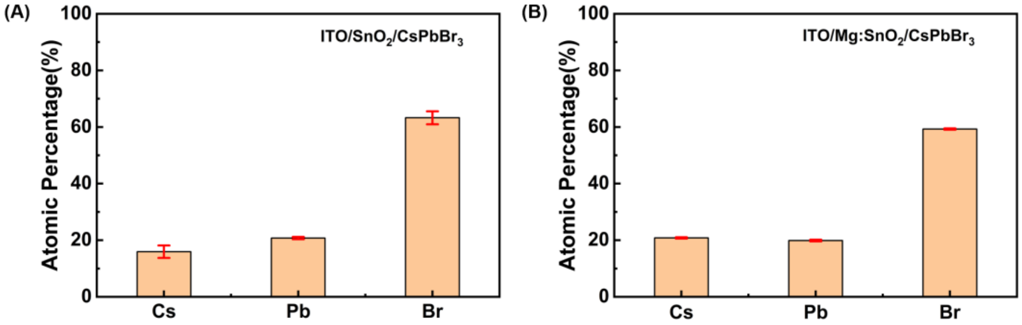

Figure S4 Histogram of atomic percentages of Cs, Pb, and Br elements in the $CsPbBr_3$ absorption layer with error bars. (A) ITO/SnO_2/$CsPbBr_3$; (B) ITO/ Mg:SnO_2/$CsPbBr_3$.Compared to the control sample, the elemental ratio of Cs:Pb:Br in the Mg-doped sample (B) is closer to the ideal stoichiometric ratio of 1:1:3, indicating improved compositional uniformity of the perovskite layer after Mg doping, related to Figure 5. Data are represented as mean ± standard deviation (SD), with n = 3.

Table S1 Comparison of atomic percentages of Cs, Pb, and Br in $CsPbBr_3$ films without and with 3.0% Mg doping, related to Figure 5.

	Cs (%)	Pb(%)	Br(%)	Total(%)
SnO_2	15.96	20.77	63.27	100
Mg:SnO_2	20.85	19.88	59.27	100

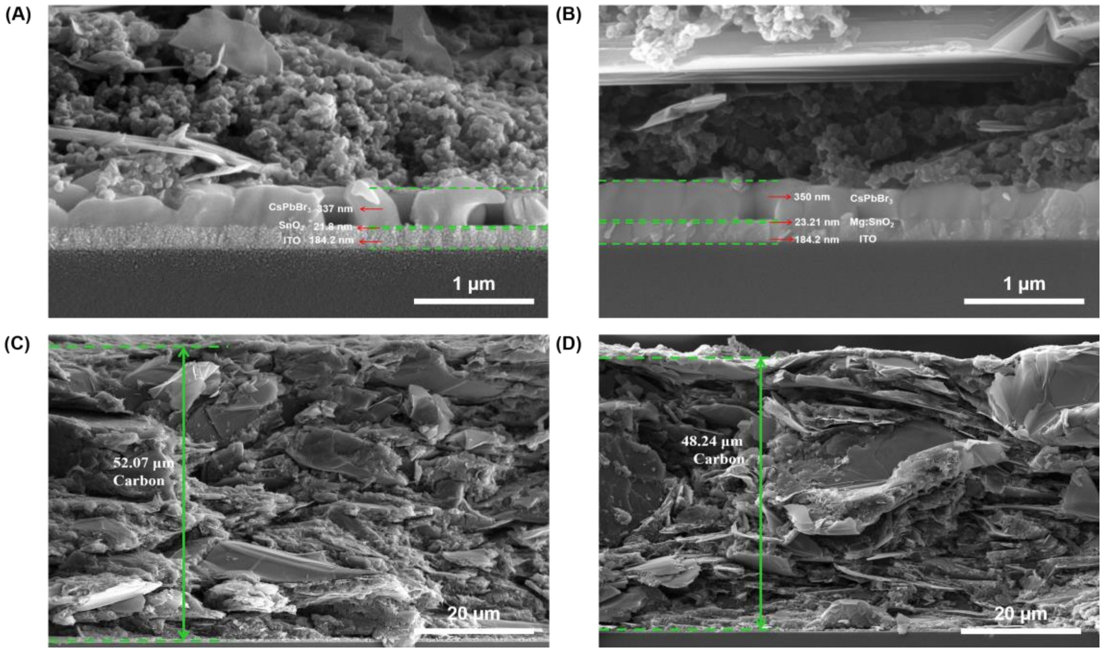

Figure S5 The thickness of device layers by Cross-sectional SEM patterns based on: (A) , (C): ITO/SnO_2/$CsPbBr_3$/Carbon and (B) , (D): ITO/3.0% Mg-doped SnO_2/$CsPbBr_3$/Carbon, related to Figure 6 and Figure 7. (A) , (B): High-resolution cross-sectional images showing the thickness of the $CsPbBr_3$ layer and the underlying layers. Scale bars: 1 μm.

(C) , (D): Low-magnification cross-sectional images highlighting the thickness of the carbon electrode. Scale bars: 20 μm.

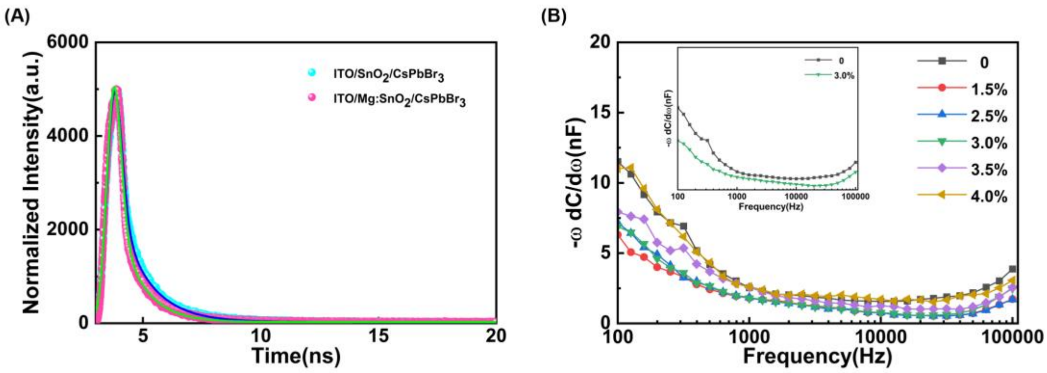

Figure S6 Comparison of transient photoluminescence (TRPL) and trap state density responses before and after Mg doping: (A) TRPL decay curves of $CsPbBr_3$ films; (B) Frequency-dependent trap state density curves of devices with varying Mg doping concentrations, related to Figure 10.

Table S2 TRPL fitting parameters of perovskite films deposited on SnO_2 and 3.0% Mg-doped SnO_2 ETLs, related to Figure 10.

Structure	τ_1(ns)	A_1(%)	τ_2(ns)	A_2(%)	τ_{avg}(ns)
ITO/SnO_2/$CsPbBr_3$	0.136	86.54	1.283	13.46	0.284
ITO/3%Mg:SnO_2/$CsPbBr_3$	0.059	94.71	1.046	5.29	0.111

Table S3 Average photovoltaic parameters of PSCs based on SnO_2 Films with different Mg doping ratio, related to Figure 11.

	PCE (%)	Voc (V)	FF (%)	Jsc ($mA{\cdot}cm^{-2}$)
0%	5.409	1.397	60.11	6.440
1.5%	6.210	1.516	62.28	6.583
2.5%	6.560	1.539	63.14	6.761
3.0%	7.174	1.540	66.98	6.962
3.5%	6.249	1.531	63.05	6.549
4.0%	5.976	1.508	60.66	6.531

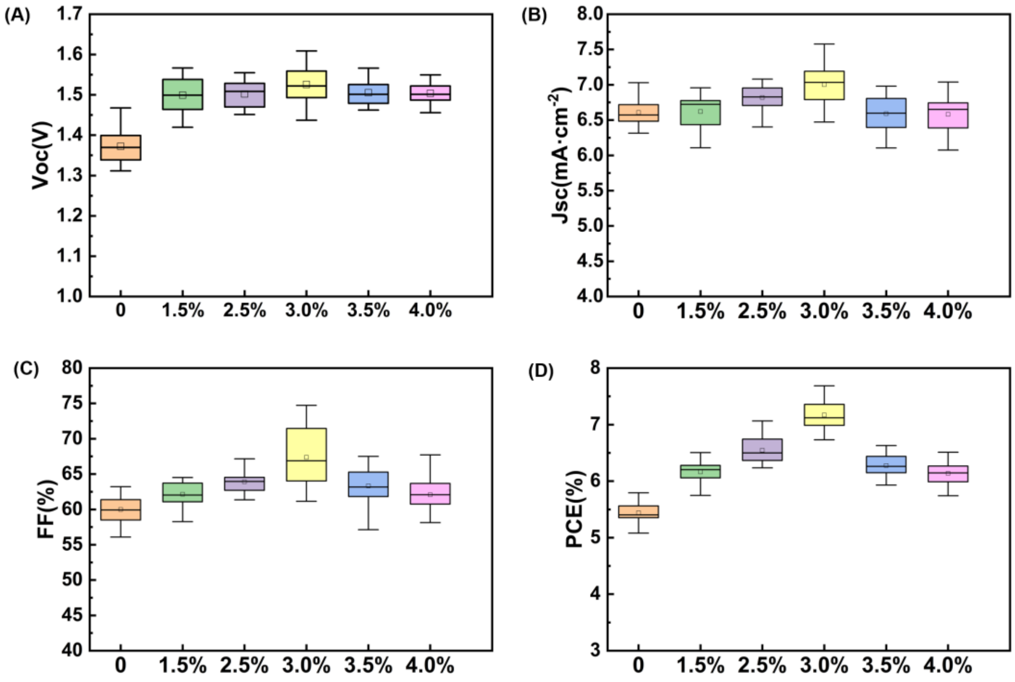

Figure S7 The parameters of devices based on 20 device samples at different Mg doping concentrations: (A) Voc, (B) Jsc, (C) FF, and (D) PCE. The plots illustrate the distribution and variation in device performance under different doping conditions, reflecting the good reproducibility and stability of the devices, related to Figure 11. Each box plot represents the interquartile range (IQR), with the center line indicating the median, and whiskers showing the minimum and maximum values.

Table S4 Reproducibility test results based on 20 device samples, showing the statistical performance parameters (PCE, Voc, FF, and Jsc) under different Mg doping concentrations, related to Figure 11.

	PCE (%)	Voc (V)	FF (%)	Jsc ($mA \cdot cm^{-2}$)
0%	5.44115	1.37235	59.99069	6.60651
1.5%	6.16275	1.49915	62.15128	6.62082
2.5%	6.54683	1.50234	63.90171	6.82129
3.0%	7.17013	1.52575	67.36011	7.00101
3.5%	6.27603	1.50543	63.33525	6.59067
4.0%	6.1368	1.50358	62.07635	6.58242

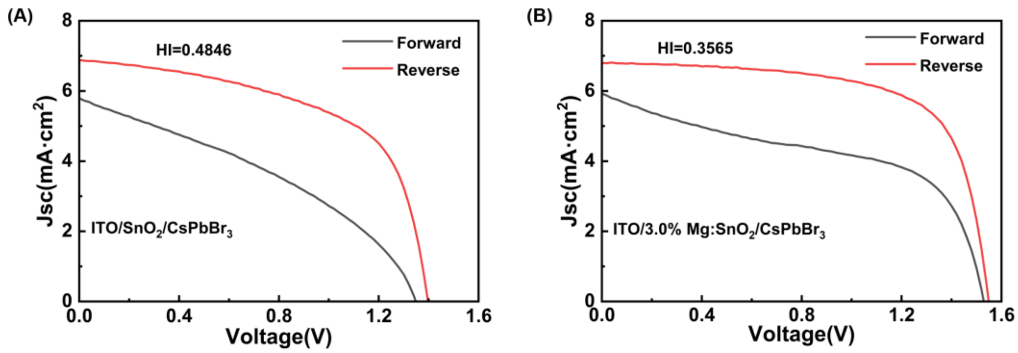

Figure S8 J–V hysteresis behavior comparison between undoped and 3.0% Mg-doped devices: (a) ITO/SnO_2/$CsPbBr_3$ device with a hysteresis index (HI) of 0.4846; (b) ITO/3.0% Mg:SnO_2/$CsPbBr_3$ device with reduced HI of 0.3565, indicating significantly suppressed hysteresis. Note:The hysteresis index is calculated as the following formula: $HI=(PCE_{Reverse}-PCE_{Forward}) / PCE_{Reverse}$. The device incorporating the Mg-doped SnO_2 layer exhibits a reduced hysteresis index (HI = 0.3565) compared to the control device (HI = 0.4846), indicating an effective suppression of the hysteresis effect due to the improved quality of the electron transport layer, related to Figure 11.

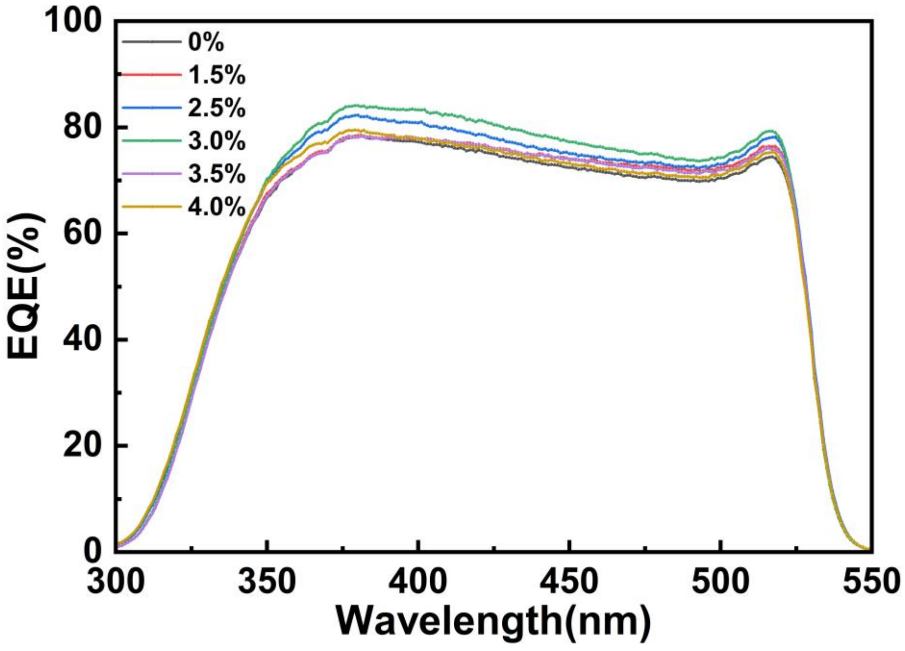

Figure S9 EQE characterizations of PSCs based on SnO_2 ETLs with different Mg doping ratio, related to Figure 12.

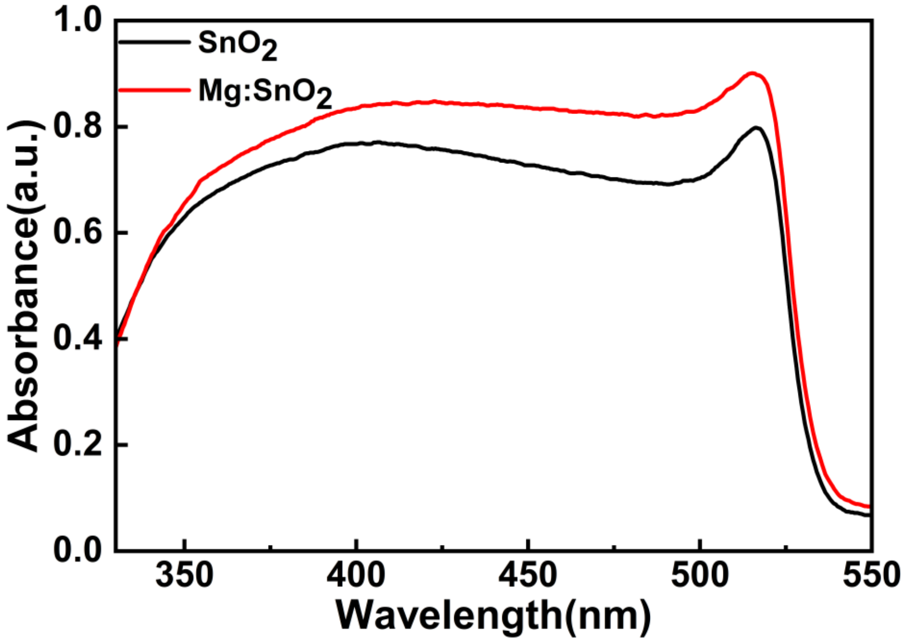

Figure S10 UV-vis absorption spectra with the construction of ITO/SnO_2/$CsPbBr_3$ and ITO/3.0% Mg-doped SnO_2/$CsPbBr_3$, related to Figure 12.
